# Exposed – a semantic concept analysis of its origin, meaning change over time and its relevance for caring science

**DOI:** 10.1080/17482631.2022.2163701

**Published:** 2023-01-02

**Authors:** Sofia Almerud Österberg, Ulrica Hörberg, Lise-Lotte Ozolins, Carina Werkander Harstäde, Carina Elmqvist

**Affiliations:** a Faculty of Health and Caring Science, Linnaeus University, Växjö, Sweden; b Department of Anaesthesiology, Kronoberg County Council, Växjö, Sweden; c R&D Department, Kronoberg County Council, Växjö, Sweden

**Keywords:** Caring science, concept analysis, exposedness, nursing care, suffering by care

## Abstract

**Purpose:**

A patient is vulnerable and exposed due to illness, relies on and surrender to other people. In caring this means a special dependency. The aim of this study was to describe the origin of the concept ‘exposed’, to elucidate how the definition of this term has changed over time, and to outline its relevance in caring science.

**Method:**

A semantic concept analysis in two phases was conducted.

**Results:**

The findings show that over time, the degree of binding of the following synonyms has become stronger; unprotected/defenseless, naked/bare, pressed/jostled and unsafe/unprotected and can easily be transferred to a clinical nursing context.

**Conclusions:**

This study provides a deeper understanding of the concept 'exposed' semantically. Being exposed is a profound experience for patients who need to be seen as the person they really are. A life-world led care has an existential power that can support professionals, strengthen patients’ health processes, and alleviate the patient’s suffering.

## Introduction

Being human includes existential vulnerability. Temporality and instability in our lives, along with the inability to control our closest living conditions, are a basis for experiencing both fragility and vulnerability in existence (Arman, 2015). To be human is to be exposed, to be a patient is to be twice as exposed, both by life and the health care system (Almerud et al., 2008). Based on this, we argue that *exposed* is a concept whose relevance for caring science should be ascertained. The term exposed (utsatt) is used in the Swedish language in various ways, e.g., for people in vulnerable positions.

Caring ethics is the basic approach towards the patient and the care, regardless of the ethical guidelines that exist for each health care profession. Human dignity, meaning the right to be confirmed as a unique person, precedes everything else. Suffering raises the ethical requirement for care (Eriksson, 2018). The ethical demand is directed towards the caregiver and emanates from the patient (Dahlberg et al., 2003). The demand is absolute, i.e., it is an ethical obligation for the caregiver directed towards meeting this demand and to reduce exposedness. Lögstrup (Lögstrup, 2009) states that trust is the ground for our existence. In the encounter with the unknown, we trust the Other and thereby trust is also the core of a caring relation. A sick person relies on and surrenders to carers and is therefore dependent, exposed and vulnerable (Almerud et al., 2008; Dahlberg et al., 2003). The goal of caring is to alleviate suffering and create conditions for well-being (Lögstrup, 2009; Martinsen, 2012). Suffering can also emanate from care. Suffering caused by care is the suffering that the health care providers cause the patient by violating the patient’s dignity, e.g., exercising power or failing in their care (Eriksson, 2018). Neglect and denial of care in health care are examples of suffering caused by carers due to insecurity and lack of information and incomprehensibility in the care environment (Dahlberg et al., 2003).

A conceptual understanding is important in search of knowledge. Eriksson (Eriksson, 2010) states that it is through concepts that reality is shaped; selection and exploration of core concepts is the first phase of knowledge formation. Concepts are labels of phenomena. They are construction parts in building theories. Through concepts, one can also understand peoples’ ways of being as well as their practical, everyday lives and professional thinking (Arman, 2015). Concept determination may thus be seen as an important component for basic caring science research (Eriksson, 2010). The theoretical ground for this paper is the concept’s relevance within caring science.

## Aim

The aim of this study was to describe the origin of the concept “exposed”, to elucidate how the definition of this term has changed over time, and to outline its relevance in caring science.

## Method

A semantic concept analysis entails an etymological description and analysis of the definitions of linguistic expressions taken from dictionaries (Eriksson, 2010; Koort, 1975; Sivonen et al., 2010). It creates an understanding of the conceptual meaning in the language of one time period at a time (Sivonen et al., 2010). Furthermore, a semantic concept analysis can help determine the ontological and contextual perspectives (Eriksson, 2010) and create a deeper understanding of a concept in a caring science context (Sivonen et al., 2010). By developing theories and concepts, caring science can move towards a deeper understanding of the essence of caring (Arman, 2015). The Koort (Koort, 1975) tradition of semantic analysis contributes to a useful exploration and definition of central concepts within caring science (Sivonen et al., 2010). The method enables a deeper and broader understanding of caring science phenomena on an ontological level, thereby offering solid ground for theoretical, contextual and practical understanding and development. Analysing the concept semantically can deepen the understanding of a person’s sense of being exposed in the context of health care (Koort, 1975; Sivonen et al., 2010).

The semantic analysis of concepts, originally created by Koort (Koort, 1975) and further developed by Eriksson (Eriksson, 2010), is a common method within Nordic caring science research to add knowledge when a concept or phenomenon needs clarification. Thus, a concept is not merely a combination of words or letters but is closely intertwined with human life and lived experiences (Eriksson, 2010; Werkander Harstäde et al., 2012). By defining concepts within specific contexts, knowledge relevant for caring science can be established (Eriksson, 2010). A semantic analysis can show a pattern of meaning, give a more complete picture of the concept and illustrate the metamorphosis of the concept over time (Nåden & Eriksson, 2003). Furthermore, a semantic analysis aims to analyse the meaning of linguistic expressions (Koort, 1975). The method includes two phases: 1) etymological and 2) semantic.

### Material, inclusion and exclusion

#### Sample

In a semantic analysis, the researcher should avoid mixing verbs, adjectives and nouns (Sivonen et al., 2010). The adjective form *exposed* was chosen as it appears in more and earlier dictionaries than the verb *expose* and the noun *exposedness*.

#### Data

The material used is dictionaries chosen to span as long a period of time as possible (Koort, 1975) to ensure that a development of the concept and its meaning and synonyms can be established. The first time *exposed* appears is in Illustrated Swedish Dictionary from 1964. The concept was examined in eight dictionaries selected based on their scientific authority (Eriksson, 2010), i.e., they had a high author competence and were published by well-known, established and high quality publishers. The dictionaries in the 2004 semantic analysis were published between 1964 and 2002 (Tables 1 and 2). To elucidate how the concept’s meaning changed over time, a new concept analysis, using the same method, was conducted fifteen years later, in 2019. Eight dictionaries, published between 2006–2017, were used (Table 2).

**Table I.** Dictionaries used in the semantic concept analysis in 2004.

Johannisson, T. & Ljunggren, K-G. (1977). Svensk handordbok: konstruktioner och fraseologi. (Swedish handbook of words: constructions and phraseology). Stockholm: Norstedt. Malmström, S. (2002). Bonniers svenska ordbok. (Bonnier’s Swedish dictionary). Stockholm: Bonnier. Molde, B. (Red.). (1964). Illustrerad svensk ordbok. (Illustrated Swedish dictionary). Stockholm: Natur och Kultur Nationalencyklopedins ordbok Bd 3. (1996). (The Dictionary of the National Encyclopaedia). Höganäs: Bra böcker. Ord för ord: svenska synonymer och uttryck. (1992). (Word by word—Swedish Synonyms and Phrases). Stockholm: Norstedt. Strömberg, A. (1988). Synonymordboken. (Thesaurus). Stockholm: Strömberg. Svensk ordbok (Swedish Dictionary). (1986). Uppsala: Norstedt. Walter, G. (2000). Bonniers synonymordbok. (Bonnier’s Thesaurus). Stockholm: Bonnier.

**Table II.** Dictionaries used in the semantic concept analysis in 2019.

Gerhardsen, H. (red.) (2009). Svenska synonymordbok. 5 uppl. (Swedish thesaurus, 5th ed.). Stockholm: Norstedt Köhler, P.O. & Messelius, U. (2006). Natur och kulturs stora svenska ordbok. (Natur och Kultur’s large Swedish dictionary). Stockholm: Natur och Kultur Nationalencyklopedin. (n.d.). Svensk ordbok. (Swedish dictionary). Höganäs: Bra Böcker (www.ne.se) Nationalencyklopedin. (n.d.). Svenska synonymer. (Swedish thesaurus). Höganäs: Bra Böcker (www.ne.se) Sjögren, P.A., Györki, I., Malmström, S. (2010). Bonniers svenska ordbok. (Bonnier’s Swedish dictionary). Stockholm: Bonnier Svenska Akademiens ordlista. (2015). 14 uppl. (Swedish Academy glossary). Stockholm: Svenska Akademien Svenska Akademiens ordbok. (2012). (Swedish Academy dictionary). Stockholm: Svenska Akademien. Svensk ordbok. (2009). (Swedish dictionary). Stockholm: Svenska Akademien.

First, an **etymological description** was made using dictionaries to indicate the concept’s age, origin and change over time. Thereafter, a **discrimination analysis** was made to investigate how the concept and its synonyms are related, by exploring possible bindings. A binding entails either that the lexical description of the concept includes the synonym, or the reverse. The discrimination analysis consists of three phases: matrix, paradigm and interpretation phase.

#### The matrix phase

To create a family of concepts, synonyms were chosen. The choice of synonyms for the concept *exposed* was formed by the strongest quantitative concept dimension *in an exposed position*. Words that seemed relevant for caring science, i.e., words related to human existence, were chosen (Table 3). How the synonyms are related to each other has not been explored in this paper.

**Table III.** Matrix over bindings and degree of synonymy to the concept”exposed”.

Synonyms to the concept EXPOSED (Utsatt)	Number of times the concept is bound to the synonym		Number of times the synonym is bound to the concept		Total number of bindings		Number of dictionaries in which the concept is found		Number of dictionaries in which the synonym is found		Degree of synonym %	
	2004	2019	2004	2019	2004	2019	2004	2019	2004	2019	2004	2019
Exposed (Utsatt)							8	8				
Precarious (Prekär)	2	0	1	0	3	0	8	0	2	0	30	0
Perilous (Riskabel)	4	3	2	2	6	5	8	8	4	8	50	31
Threatened (Hotad)	2	2	1	0	3	2	8	8	2	1	30	22
Dangerous (Farlig)	3	3	1	2	4	5	8	8	3	6	36	36
Vulnerable (Blottställd)	3	3	3	2	6	5	8	8	3	5	55	38
Unprotected/Defenceless (Oskyddad)*		2		2		4		8		4		33
Naked/Bare (Blottad)*		2		1		3		8		3		27
Pressed/jostled (Trängd)*		2		2		4		8		4		33
Unsafe/unprotected/exposed (Exponerad)*		3		1		4		8		2		40

Note: *In 2004, these synonyms were only discovered in one dictionary and they were therefore not chosen for the analysis. In 2019, however, the binding is strong.

##### The paradigm phase

**Figure 1.**
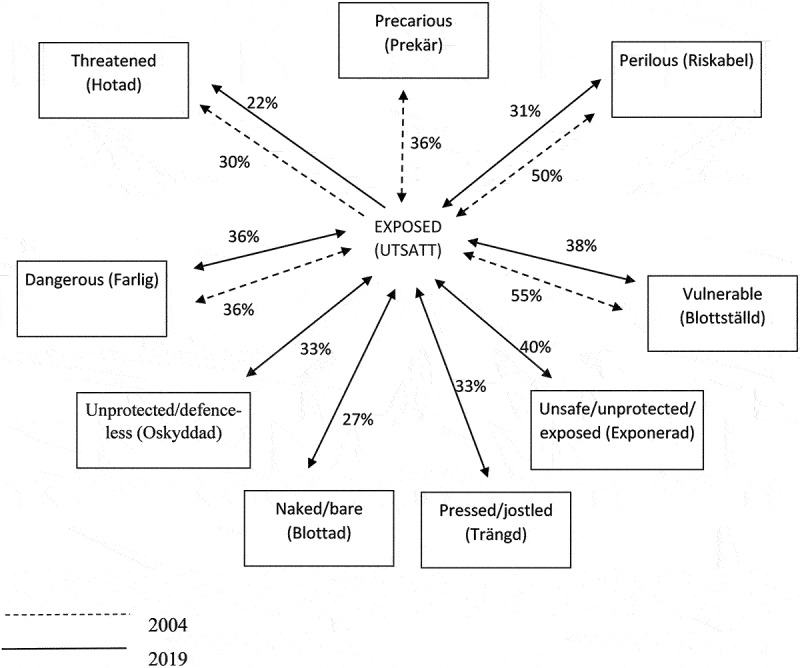
Discrimination paradigm for the concept exposed between 1964–2017. The numbers correspond with degrees of synonymy in percent. Links are illustrated by arrows. The words within brackets are the Swedish words.

consists of the construction of a discrimination paradigm (Figure 1). This shows the relation between the concept and its synonyms (Koort, 1975). The heading “Degree of synonymy between two words” means the concordance in percent according to the chosen dictionaries; this enables the understanding and interpretation of dimensions within the family of concepts (Koort, 1975). If the degree of synonymy is lower than 50%, it can be questioned whether the word is an actual synonym. However, this can be interpreted as a nuance of meaning (Eriksson, 2010; Koort, 1975). For the concept *exposed*, relevant synonyms, regardless of the degree of synonymy, were chosen if they were regarded relevant for caring science, which is the context of the aim of this study. The degree of synonymy, expressed in percent, was calculated according to Koort’s (Koort, 1975) formula:

(existing binding + existing binding) x 100/possible binding + possible binding

For example, exposed (utsatt) is bound to the synonym perilous (riskabel) in four of the eight dictionaries; perilous (riskabel) is bound to the concept exposed (utsatt) in two of these dictionaries. The concept exposed (utsatt) is an entry in all eight dictionaries; the synonym perilous (riskabel) is an entry in four of these dictionaries. The degree of synonymy is calculated according to the formula: (4 + 2)×100/8 + 4 = 50%.

## Ethical considerations

All research, empirical or theoretical, raises ethical questions. By striving for as open and well-founded argumentation as possible, we have strived to follow the directions for good scientific practice, claiming both transparency and stringency (2004, 2013). Sivonen et al (Sivonen et al., 2010) highlights areas to be considered while conducting a semantic concept analysis. A reflective process guided the analysis by striving to clearly show what choices were made in both data gathering and analysis. When choosing dictionaries, the aim was to gather a broad and wide source of materials to avoid a narrow perspective.

## Results

The results start with an etymological and historical description of the concept followed by a matrix phase where dimensions of meaning and synonyms are presented. The paradigm phase shows how the different synonyms are related to the concept, and the concept’s change over time is described.

### Etymological and historical description

Bring (Bring, 1930) does not address the word *exposed* separately, but refers to the hypernym “unfree/not free” where, for example, *exposed to* and *vulnerable* are listed as synonyms (cf. discrimination matrix, Table 2). Almhult (Almhult, 1955) describes the meaning of *exposed* as “post or position exposed to danger or criticism”. *Exposedness* as a noun does not exist in older dictionaries, e.g., the word exists in the Swedish Academy glossary, 11th edition (1986), but not in the 10^th^ edition (1973). However, the adjective form *exposed* exists in both. The Dictionary of the National Encyclopedia (Nationalencyklopedin, N.d.) states that the adjective form *exposed* has been used in text since 1553.

### Matrix phase

The word “exposed” (utsatt), in its adjective form, existed in most of the selected dictionaries in three different meaning dimensions (Table 4).

**Table IV.** Matrix phase.

Exposed (utsatt) – adjective form with three different meaning dimensions
With exposed name (Med utsatt namn) On scheduled time (På utsatt tid) In an exposed situation (I utsatt läge) – the focus in this study

“Which can easily be attacked” and “which is a target of attack” are two expressions that the dictionaries specify as synonymous for exposed (utsatt). However, the word attack does not appear as a synonym. This meaning shows that the person who is exposed is passive and exposed by someone else. It is interesting to note the expression “put yourself at risk” (1973). Thus, it is the subject who is active and no one else creates the exposedness. In more recent dictionaries, no distinction is made between who exposes whom; the synonyms are listed without specifying the active part.

In the concept analysis, synonyms from the dimension “in an exposed situation”, with at least two bindings to the concept were included in the first calculation of degree of synonymity, and synonyms with at least a 27% degree of synonymy were included in the subsequent analysis. The synonym “threatened” had only a 22% binding. However, this synonym was chosen in the 2004 analysis, with a 30% binding, and therefore it was also included in 2019. The synonyms are: precarious (*prekär*), perilous (*riskabel*), threatened (*hotad*), dangerous (*farlig*), vulnerable (*blottställd*), unprotected/defenceless (*oskyddad*), naked/bare (*blottad*), pressed/jostled (*trängd*) and unsafe/unprotected/exposed (*exponerad*).

### Paradigm phase

In the discrimination paradigm (Figure 1) the synonyms are presented in relation to the concept. For descriptions of the synonyms, see Table 5.

**Table V.** Paradigm phase.

Precarious (prekär), adjective.	Entry in only two of the chosen dictionaries from 2004. A double binding between the concept and the synonym. In 2019 the synonym is no longer found in any dictionaries.
Perilous (riskabel), adjective.	Entry in four of the chosen dictionaries from 2004 and in all eight of the chosen dictionaries from 2019. A strong initial binding that weakened between 2004 and 2019.
Threatened (hotad), adjective.	Entry in only two of the chosen dictionaries from 2004 and one from 2019. The only synonym that has a single binding from the concept, but not back from the synonym to the concept. The binding has weakened between the two concept analyses.
Dangerous (farlig), adjective.	Entry in three chosen dictionaries from 2004 and in six chosen dictionaries from 2019. A moderately strong binding that is stable between 2004 and 2019.
Vulnerable (blottställd), adjective.	Entry in three chosen dictionaries from 2004 and in five chosen dictionaries from 2019. The synonym with the strongest degree of binding. However, the binding weakened from 55% to 38% between 2004 and 2019.
Unprotected/defenceless (oskyddad), adjective.	The synonym was only found in one chosen dictionary from 2004 and had a weak binding. Therefore, the synonym was not chosen for the original analysis. In 2019, however, the entry was in two of the chosen dictionaries and the binding was moderate.
Naked/bare (blottad), adjective.	The synonym was only found in one of the selected dictionaries from 2004 and had a weak binding. Therefore, the synonym was not chosen for the original analysis. In 2019, however, the entry was in all eight of the chosen dictionaries and the binding was moderate.
Pressed/jostled (trängd), adjective.	The synonym was only found in one of the chosen dictionaries in 2004 and had a weak binding. Therefore, the synonym was not chosen for the original analysis. In 2019, however, the entry was in four of the chosen dictionaries and the binding was moderate.
Unsafe/unprotected/exposed (exponerad), adjective.	The synonym was only found in one of the chosen dictionaries from 2004 and had a weak binding. Therefore, the synonym was not chosen for the original analysis. In 2019, however, the entry was in two of the chosen dictionaries and the synonym had the strongest degree of binding (40%).

### Metamorphoses over time

*Precarious* does not appear as a synonym in dictionaries published after 2006. The one synonym that is stable regarding its degree of binding is Dangerous. Precarious, Perilous and Threatened have weakened. Synonyms that have become stronger bindings are unprotected/defenceless, naked/bare, pressed/jostled and unsafe/unprotected/exposed (Table 6).

**Table VI.** The lexical change of the concept over time (2004–2019).

	Degree of binding in 2004	Degree of binding in 2019	Change from 2004-2019
Precarious (Prekär)	36	Synonym not found	Disappears as a synonym
Perilous (Riskabel)	50	31	Weaker degree of binding
Vulnerable (Blottställd)	55	38	Weaker degree of binding
Dangerous (Farlig)	36	36	Same degree of binding
Threatened (Hotad)	30	22	Weaker degree of binding
Unprotected/Defenceless (Oskyddad)	*	33	Stronger degree of binding
Naked/Bare (Blottad)	*	27	Stronger degree of binding
Pressed/jostled (Trängd)	*	33	Stronger degree of binding
Unsafe/unprotected/ exposed (Exponerad)	*	40	Stronger degree of binding

Note: * Only discovered in one dictionary in 2004 and was therefore not chosen for the analysis. In 2019, however, the binding is stronger.

## Discussion

### Of methods

A concept analysis gives definitions to the use of a concept in both theory and research and can also help clarify terms in nursing that have become catch phrases and hence either have lost or changed their meaning (Eriksson, 2010; Sivonen et al., 2010). In this concept analysis of the word exposed, we chose to further investigate the meaning dimensions *in an exposed situation*, since this meaning is most related to clinical caring practice. This may be seen as a limitation. Perhaps a broader view of the concept might have given a different understanding but our results show a robust description of the concept related to caring. To ensure a robust analysis of the concept exposed and determine its relevance for a caring science, a semantic analysis was conducted to get a sense of the meaning of the concept. To elucidate how the term might have changed over time, the analysis was made twice, with a 15 year gap in time. In the analysis from 2004, only printed books were used. Since then, digital sources have become available. Thus, in the second analysis, digital as well as printed sources were used.

Koort (Koort, 1975) states that the degrees of synonymy give a picture of the relationship between synonyms and concepts. A degree of synonymy below 50% may be questionable in terms of relevance and the meaning of the concept can become too vague. On the other hand, weaker synonyms can constitute a nuance of meaning, which is important for the understanding of the concept (Nåden & Eriksson, 2003). In this study, synonyms regardless of the degree of synonymy, were chosen if the authors regarded them as relevant for caring science. Nuances of a concept might be valuable while trying to understand a concept in a complex context such as human existence. All authors have long and both broad and deep knowledge in caring science.

The choice of language may be considered as a limitation. However, Sivonen et al (Sivonen et al., 2010). states that a semantic concept analysis should preferably be carried out in the native language of the researcher. However, we consider the knowledge generated to be of wider common interest and therefore wanted to create a semantic understanding of the concept with relevance to clinical caring practice in a broader and international perspective. The semantic part of the analysis was made in Swedish and translated into English. Translation has been challenging and great effort has been made to ﬁnd English words that correspond closely to the meaning content of the original Swedish words. Presenting the analysis in English was important to reach as many readers as possible. A linguistic expert has helped in proofreading the text. When difﬁculties occurred, discussions about the different alternatives took place among the authors resulting in that, on some occasions, two English words were chosen when no speciﬁc word corresponded sufﬁciently with the Swedish word.

### Of results

#### Origin of the concept and changes over time

Over the last fifteen years, the concept’s relevance for clinical caring practice has strengthened. The degree of binding of the synonyms has become stronger for those synonyms that easily can be transferred to a clinical nursing context i.e., unprotected/defenceless, naked/bare, pressed/jostled and unsafe/unprotected (c.f. Figure 1 and Table 4). In the second semantic analysis (in 2019) it became obvious that the concept *exposed* had become stronger and more stringent in the meaning dimension *in an exposed situation*.

#### Relevance for caring science

A semantic analysis can be further developed by connecting theoretical and clinical caring science from specific or more general caring contexts. The results can thereby create a deeper understanding of the data, support the analysis and further develop the understanding of the concept and the experiences in a caring context (Eriksson, 2010; Werkander Harstäde et al., 2012).

Overall, the findings (Figure 1) can be related to exposedness as an existential vulnerability (Arman, 2015). The patient role involves vulnerability, a sick person relies on and surrenders to other people. In care, this implies a special dependency, as a patient is *vulnerable* and *exposed* due to illness. This vulnerability also affects the next-of-kin (Dahlberg et al., 2003). The caregivers also have a vulnerable position. Cuts in care, staff shortages and a feeling of not being sufficient lay a foundation for stress problems and can have consequences for the quality of care. The phenomenon of vulnerability thus affects three parties: the patient, the caregiver and related persons.

From a caring science perspective, our findings showed that the concept exposed needs to be highlighted, as it entails patients suffering both from poor health and from the care they receive or do not receive, which in turn can be related to loss of dignity. Objectification of human beings in health care settings is an aspect of being *naked* and *exposed*. Being exposed means being *unprotected* as a human being in a vulnerable situation (Todres et al., 2009). The authors (Todres et al., 2009) describe a conceptual framework for a humanizing care which can be understood in relation to the concept dehumanization and describe a continuum between these dimensions and the huge difference between being seen as a subject (insiderness) or as an object. There is a continuum between, for example, togetherness and isolation, sense-making and loss of meaning, sense of place and dislocation, embodiment and reductionist body, and this points to how care should be provided to support human dignity, wellbeing and prevention or alleviation of suffering (ibid).

Maintaining human dignity means maintaining the right to be confirmed as a unique person. The presence of suffering and *vulnerability* raises the human ethical obligation to care for a unique person. The power lies in the hands of carers or of the health care system that risks the patient’s dignity. Patients can be denied alleviation of their suffering as persons and human beings, and feelings of worthlessness can arise. The power is related to lack of knowledge, the patients’ insecurity and not being understood. This exposedness means a vulnerability that can be experienced as overpowering and annihilating (Dahlberg, 2018; Eriksson, 2006).

There is a strong relationship between existence and caring. Carers ought to become much more attuned to the importance and value of the experience of the person who is suffering (Galvin & Todres, 2011). Galvin and Todres (Galvin & Todres, 2015) describe human vulnerability as “Honour-Wound”, a metaphor utilized in a poetic way to describe both possibilities and existential and literal vulnerabilities. Further, the “wound” nuance is related to terms such as *vulnerability*, *exposedness*, finitude, frailty, unprotectedness, *insecurity* etc. According to Zaner (Zaner, 2000), clinical encounters with interpersonal relationships constitute specific moral aspects and challenges. Critical issues of illness experiences can be related to dialogue, trust, *vulnerability*, *being pressed/jostled*, violence, and power, leading to *precarious* situations for the person’s own being that is being *threatened*.

Suffering caused by care is the suffering that the health care providers cause to the patient by violating the patient’s dignity, for example exercising power or failing in their care (Eriksson, 2006), thereby creating an exposedness of the patient. Furthermore, dignity can be painfully ruptured, in *danger* and in need of restoration. Alas, some degrees of rupture are very difficult, perhaps impossible, to restore (Galvin & Todres, 2015).

Cubellis (Cubellis, 2018) describes care wounds as a result of precarious vulnerability and potential exposure. The care can wound those who offer it (the carers) in a rigid and unfeeling system. It can wound when it demands exposure of the carer’s own hurt and when exposure is seized on as a means to mitigate the fissures in a larger broken system. This can mean increased exposure and suffering for the patients, who need to be handled as more than objects. Vulnerability and suffering can be sensitive issues for carers as they can be like a “sore point” that both can serve as an eye-opener and can cause “blind spots” (Thorup et al., 2012). Suffering and vulnerability, like sore points, can form the caregivers’ courage in care situations. Courage expresses itself as the courage to help patients face their vulnerability and suffering. It also bears witness to patients’ vulnerability and suffering. It also means that the carers have faith in themselves and can argue for and provide professional care. This can prevent patients’ feelings of being exposed. Todres, Galvin and Dahlberg (Todres et al., 2014) put forward the important concept of openheartedness, along with the help of life-world knowledge, as an interpretative framework for the care and understanding of others through the complex self. In care and understanding, the dialogic act is a dialectic between vulnerability and power, an immense hope and a profound risk. The hope for help in the form of cure and/or alleviated suffering and comfort are at hand. Furthermore, in every dialogue there is vulnerability, and one aspect is related to the questioner’s existential situation and the openness to a form of vital not-knowing or inability to know. The vital need is to understand and to be understood and also to know and be known in order to overcome the experiential exposedness that is intertwined with the illness (Galvin & Todres, 2015). This can be related to Dahlberg (Dahlberg, 2018) and Todres, Galvin and Dahlberg (Todres et al., 2014), who emphasize the need to be aware of the art of understanding in care. Understanding of the other can never be absolute but, in caring, one can always strive for insiderness, to fully understand the other. More often, reaching for insiderness can be experienced as more important than knowledge of details of another person such as a patient. An overreliance on the body-object gaze may shift attention away from insiderness. Thus, in care, a shift of focus is needed from mere bodily behaviour or symptoms to focus on what the living body is trying to say about the person’s insiderness. Wiklund (Wiklund Gustin, 2017) addresses the importance of the caregiver as being emotionally engaged with the patient’s stories and experiences, as opposed to being emotionally passive or distant. Leget (Leget, 2013) states that to listen, open minded, to vulnerable people is an expression of respect and attentiveness, and points to the interrelation of subjective and relational dignity. Dignity is then constituted and upheld by people interrelated in caring relationships. This has been described as a dignity experience and gives a sense of one’s interpersonal value and worthiness. When a sense of valuing the interpersonal exists, individuals are participating in an interpersonal world of mutuality (Galvin & Todres, 2015). This means a gift to one another, being intrinsically important to one another, being in community in mutual ways. According to Dahlberg (Dahlberg, 2018), just to be open to the possibility of others’ experiential worlds has profound ethical meaning within a caring context. The challenge is to empower understanding in such a way that the manifold of nuances of the life-world are displayed and come into play.

## Conclusion

Exposedness is a profound experience for patients. This concept analysis can help in deeper understanding a person’s unique exposed situation and adds to the knowledge base of exposedness within nursing care. Life-world led care has existential power that can support professionals in strengthening patients’ health processes. By this is meant finding balance as opposed to being exposed and experiencing human vulnerability. This balance alleviates the patient’s suffering and reduces the risk of suffering by care. Since caring science as a science is not connected to one specific profession, everyone who meets exposed human beings in their daily work, e.g., all health care professionals, teachers, policemen, social workers, can use this knowledge to further understand exposedness from a human perspective.
